# Getting the message straight: effects of a brief hepatitis prevention intervention among injection drug users

**DOI:** 10.1186/1477-7517-6-36

**Published:** 2009-12-15

**Authors:** Lauretta E Grau, Traci C Green, Merrill Singer, Ricky N Bluthenthal, Patricia A Marshall, Robert Heimer

**Affiliations:** 1 Department of Epidemiology and Public Health, Yale University School of Medicine, New Haven, CT, USA; 2 Center for Health, Intervention, and Prevention (CHIP) University of Connecticut 2006 Hillside Road, Unit 1248 Storrs, CT 06269-1248, USA; 3 Department of Anthropology, University of Connecticut, Storrs, CT 06269-2176, USA; 4 Health Program and Drug Policy Research Center, RAND, Santa Monica, CA, USA; 5 Department of Medical Humanities, Case Western Reserve University School of Medicine, Cleveland, OH, USA

## Abstract

To redress gaps in injection drug users' (IDUs) knowledge about hepatitis risk and prevention, we developed a brief intervention to be delivered to IDUs at syringe exchange programs (SEPs) in three US cities. Following a month-long campaign in which intervention packets containing novel injection hygiene supplies and written materials were distributed to every client at each visit, intervention effectiveness was evaluated by comparing exposed and unexposed participants' self-reported injection practices. Over one-quarter of the exposed group began using the novel hygiene supplies which included an absorbent pad ("Safety Square") to stanch blood flow post-injection. Compared to those unexposed to the intervention, a smaller but still substantial number of exposed participants continued to inappropriately use alcohol pads post-injection despite exposure to written messages to the contrary (22.8% vs. 30.0%). It should also be noted that for those exposed to the intervention, 8% may have misused Safety Squares as part of pre-injection preparation of their injection site; attention should be paid to providing explicit and accurate instruction on the use of any health promotion materials being distributed. While this study indicates that passive introduction of risk reduction materials in injection drug users through syringe exchange programs can be an economical and relatively simple method of changing behaviors, discussions with SEP clients regarding explicit instructions about injection hygiene and appropriate use of novel risk reduction materials is also needed in order to optimize the potential for adoption of health promotion behaviors. The study results suggest that SEP staff should provide their clients with brief, frequent verbal reminders about the appropriate use when distributing risk reduction materials. Issues related to format and language of written materials are discussed.

## Background

The "Diffusion of Benefit through Syringe Exchanges Programs" (DOB) project, a longitudinal study of 584 active injection drug users (IDUs) in Chicago, IL, Hartford, CT, and Oakland, CA, used chain referral, ethnographic methods, semi-structured interviews, and mark-and-recapture techniques to measure diffusion of risk reduction supplies and messages from syringe exchange programs (SEPs) into the larger IDU community. Preliminary findings from the main study indicated that many IDUs improperly stanched their blood flow post-injection, and SEP clients were especially likely to misuse alcohol pads for this purpose [1]. Since alcohol inhibits coagulation, post-injection use increases the volume of spilled blood and leaves wound sites open to potential infection (e.g., hepatitis B and C). Based upon these findings, we developed a brief hepatitis prevention intervention. The intervention was easily integrated into existing SEP operations with minimal staff burden. The current sub-study assesses whether injection hygiene messages included in this brief intervention were associated with exposure to the intervention and whether the intervention messages and materials diffused beyond the point of distribution and into the larger IDU community.

## Methods

We conducted the hepatitis intervention sub-study in Year 2 of the four-year longitudinal study. The study design for the main study, methods, population characteristics, and injection-associated behaviors are described elsewhere [1-3]. Recruitment for the main study was via snowball sampling, initiating at the SEPs, and we were thus able to subsequently categorize participants according to the extent of their SEP use. Participants were interviewed away from SEP sites, most typically on the streets or at DOB storefronts. They were administered a semi-structured interview that assessed pre- and post-injection practices and familiarity with and use of the SSs. The analyses reported herein are based solely on the self-reported data collected from the 208 DOB participants successfully contacted and interviewed for this sub-study.

**Figure 1:**
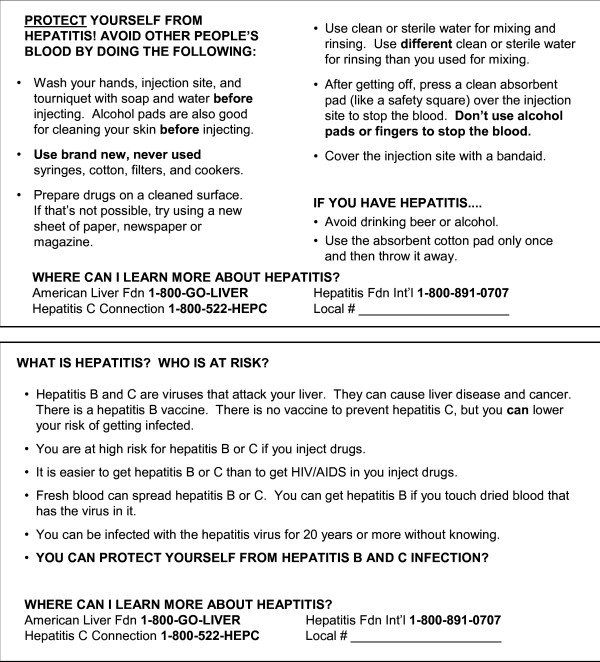
**The palm card distributed with a packet of Safety Squares at the intervention sites**.

The intervention packets consisted of ten small absorbent pads to be used instead of alcohol pads for post-injection stanching of blood. These were not commercially available, and we named them "Safety Squares" (SSs) to connote that their use could reduce injection-associated risk. The packets also included a palm card (Figure 1) about hepatitis risk, injection hygiene, and information about additional resources. It was written at a 10th-grade reading level (approximately equivalent to that of *Sports Illustrated *or *Newsweek*). SEP staff distributed the intervention packets to every client visiting the exchange for one month. Staff were not specifically prohibited from discussing the materials with clients, but given the limited time and staffing, it is estimated that less than 10% of clients spoke with staff about risk reduction and even less about the hepatitis intervention; conversations were typically very brief (e.g., "Use alcohol pads before injecting and Safety Squares after").

Following the month-long distribution period, DOB participants were contacted in the field and shown SSs prior to administering the hepatitis intervention survey. Those reporting that they had seen one previously and correctly identified its purpose were classifed as exposed to the intervention; those incorrectly identifying the SSs or reporting never having seen it before were considered 'unexposed'.

This sub-study is a secondary analysis of self-reported data, and we limited our analyses to bivariate analyses only (Chi-square tests, Fisher's exact tests, t-tests) to examine differences between exposed and unexposed participants on sociodemographics and the two injection hygiene messages targeted in the intervention (i.e., pre-injection cleaning and post-injecting stanching practices).

## Results

A total of 208 participants were interviewed between June 2000 and July 2001. The larger DOB cohort and the sub-study sample were comparable across sociodemographic and drug use characteristic except that the sub-study group had significantly (p < 0.05) fewer Whites (7.2% vs. 23.7%), more African Americans (55.3% vs. 33.8%), and fewer self-reported histories of hepatitis C infection (12.9% vs. 87.1%). There were also more participants from Chicago (57.2% vs. 45.2%) and Hartford (30.8% vs. 22.1%) and fewer from Oakland (12.0% vs. 32.7%) than in the larger DOB cohort.

The sub-study sample was 59.6% male, 92.8% from racial/ethnic minority groups, and predominantly heroin injectors. The mean age was 41.4 years (SD 9.0), and 12.2% had less than a 9^th^-grade education. On average, participants had been injecting for 18 years (SD 11.1). In the previous month, the mean number of injections was 86.9 (SD 69.4), mean injections per syringe was 6.1 (SD 12.6), and 20.9% of participants reported receptive syringe sharing at least once within the previous 30 days. No differences were noted in injection behaviors between the exposed and unexposed groups in the above injection characteristics.

Compared to the unexposed group, participants who had seen the SSs were significantly more likely to use SEPs (57.1% clients vs. 38.2% non-clients; - < 0.01); Exposure groups also differed with respect to city, and race/ethnicity (Table 1). Logistic regression analysis revealed that the likelihood of intervention exposure was greater in Hartford and for Hispanics.

**Table 1:** Characteristics of Safety Square intervention participants

Variable	Unexposed to intervention *n = 118 (%)*	Exposed to Intervention *n = 90 (%)*	Total *n = 208 (%)*	Test statistic, p-value
Site				15.15, p < .001
Chicago	81 (68.6)	38 (42.2)	119 (57.2)	
Hartford	25 (21.2)	39 (43.3)	64 (30.8)	
Oakland	12 (10.2)	13 (14.4)	25 (12)	

Age [mean (SD)]	41.8 (8.5)	41.0 (9.7)	41.4 (9.0)	NS

Female	48 (40.7)	36 (40.0)	84 (40.4)	NS

Race/ethnicity				
Non-Hispanic White	10 (8.5)	5 (5.6)	15 (7.2)	17.29, p < .001*
African American	78 (66.1)	37 (41.1)	115 (55.3)	
Hispanic	29 (24.6)	45 (50.0)	74 (35.6)	
Other/not reported	1 (.8)	3 (3.3)	4 (1.9)	

Less than high school education	52 (44.4)	52 (57.8)	104 (50.2)	NS

Earning <$1000 per month	77 (65.2)	60 (66.7)	137 (65.9)	NS

SEP user	24 (20)	32 (35.6)	56 (26.9)	6.0, p = .01

Drug treatment (Ever)	83 (71.6)	70 (79.5)	153 (75)	NS

Ever tested for HIV	82 (92.1)	108 (92.3)	190 (92.2)	NS

Diagnosed HIV+	16 (14.7)	11 (13.3)	27 (14.1)	NS

History of				
Hepatitis B	15 (13)	11 (13.1)	26 (13.1)	NS
Hepatitis C	11 (9.5)	15 (17.4)	26 (12.9)	NS
Hepatitis of unknown etiology	9 (7.9)	14 (15.6)	23 (11.3)	NS

Hepatitis B vaccinated (ever)	31 (27.7)	22 (27.8)	53 (27.7)	NS

Heroin as the drug injected most often in past 30 days	80 (67.8)	66 (73.3)	146 (70.2)	NS

* Exposure associated with Hispanic ethnicity p = .06 (reference = Non-Hispanic white and all other groups)

Pre-injection hygiene appeared to be generally adequate, with most participants stating that they usually used alcohol pads (92.5%) or water (32.5%) to clean their injection site. It is of note that 8.1% of the exposed group also reported using SSs at pre-injection despite written instructions to use them only at post-injection. It was unclear whether SSs were used to clean the skin with alcohol or soap and water, although it seems unlikely as these items were rarely observed during ethnographic activities associated with the main study.

**Figure 2:**
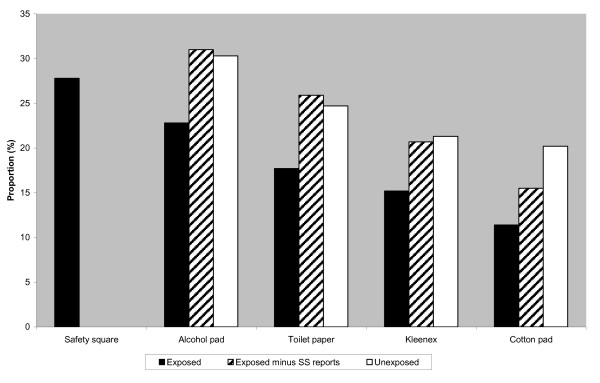
**Proportion of participants reporting their method of stanching blood post-injection by exposure group**. *Exposed *refers to study participants who reported having seen a Safety Square and correctly indentified its purpose. *Exposed minus SS reports *refers to study participants in the exposed group not reporting Safety Squares as the most frequent material used to stanch blood post-injection. This group represents people exposed to the intervention who may exhibit behavior change short of primary reliance upon Safety Squares. *Unexposed *refers to study participants who incorrectly identified the Safety Squares or reported never having seen it before.

With respect to post-injection hygiene, 27.8% of those exposed to the intervention reported usually using the SSs to stop the flow of blood (Figure 2), suggesting that the intervention was effective in reaching and changing behavior in at least one-quarter of participants. However, regardless of exposure condition, alcohol pads continued to be commonly mentioned for stanching blood post-injection (22.8% of exposed vs. 30% of unexposed; p > 0.05).

Finally, we assessed the effectiveness of prevention messages contained in the palm card. Several of these messages were consistent with other messages emanating from the SEPs (e.g., use of clean cookers and water and the washing of hands). These three messages were all but uniformly endorsed by both exposed and unexposed groups (clean cookers: exposed = 91.3%, unexposed = 95.8%; clean water: exposed = 92.5%, unexposed = 95.8%; clean hands: exposed = 87.5%, unexposed = 91.7%). In contrast, the exposed group was less likely to endorse messages about improving injection hygiene via pre-injection cleaning of tourniquets (38.8% vs. 59.2%; Χ^2 ^4.74, p = .03), drug preparation surfaces (72.5% vs. 84.4%; Χ^2 ^3.71, p = .03), or clothes (51.3% vs. 77.1%; Χ^2 ^12.9, p < .0001).

## Discussion

Over one quarter of those exposed to the intervention reported adopting use of SSs, providing preliminary evidence that low-intensity interventions can benefit the public health. Although those who were exposed to the intervention were significantly more likely to be SEP clients, non-client exposure rates suggested that some diffusion of the intervention occurred into the larger IDU community.

However, the accompanying written material was not so well received. The palm card's 10^th ^grade reading level may have been too high. Although we know of no assessments of reading levels among IDU populations, one possibility is that the palm card was not easily comprehended. The readability scores of other materials designed for IDUs have generally been lower, around 7th grade [4]. It may be that the formatting of printed materials may be as important as their content. Inappropriate use of SSs may have been less likely to occur if the message had appeared earlier in the palm card and/or the number of unique messages had been fewer.

The findings also suggest that, while passive distribution of risk reduction materials may be associated with subsequent adoption of healthier behaviors, such campaigns may not be sufficient if not accompanied by frank dialogue and explicit instructions about appropriate use of these materials. Injectors may be unaware of the anticoagulation property of alcohol, instead believing that post-injection swabbing with alcohol protects against blood-borne infections. SEPs routinely distribute health promotion brochures, and customers may become inured or confused by the plethora of information. Communication problems continue to plague HIV risk reduction efforts: condom misuse persists despite package inserts and condom use campaigns [5,6]. Our findings are consistent with reports of unintended consequences of SEP-based HIV prevention activities [7] and suggest that, along with continued distribution of risk reduction materials, risk reduction staff should be encouraged to instruct their clients - briefly and often - about the proper use of these materials.

Study limitations such as non-random sampling and social desirability factors may reduce the potential generalizability of the findings. However, at the time of the hepatitis intervention sub-study our research staff had long-standing relationships (>1 year) with participants, having observed and interacted with them away from the SEPs; social desirability factors are therefore considered to be minimal, and differences in exposure across the three sites could be due to structural differences among SEPs such as location, hours of operation, legal status, between-city differences in the dynamics of injecting drug use, syringe availability, or other reasons [2]. It is also possible that the Safety Square and the palm card could have been uncoupled. This may have resulted in some participants having been exposed to only one part of the intervention (i.e., the Safety Square). We therefore have limited evidence to suggest that the intervention worked, and a more rigorously verifiable definition of exposure would be warranted in any large scale evaluation of intervention efficacy.

In conclusion, the brief intervention that was implemented in this study resulted in a modest uptake by the injecting community, and intervention messages and materials diffused beyond the SEPS from which they originated. However, when developing written material, its content and format should be carefully reviewed and beta-tested to ensure that it is clear, understandable, and acceptable to targeted audience. Finally, while these written materials are useful in disseminating harm reduction messages, they should never supplant frank and open discussions of harm reduction strategies with end-users.

## Competing interests

The authors declare that they have no competing interests.

## Authors' contributions

LEG led the writing, was responsible for overseeing data collection and data management and synthesis of the analyses. TCG was responsible for data management, conducted the analyses, and reviewed drafts of the synthesized the analyses. RNB, PAM, and MS helped develop the intervention, were responsible for the conduct of this study at their respective sites, and reviewed drafts of the manuscript. RH conceived of the study and supervised all aspects of its implementation. All authors read and approved the final manuscript.
